# Multi-omics and machine learning reveal LYZ and ISG15 as diagnostic and therapeutic targets in autoimmune-mediated chronic kidney disease

**DOI:** 10.3389/fimmu.2026.1922463

**Published:** 2026-09-01

**Authors:** Haofeng Zheng, Jieyi Dong, Qingfu Dai, Wangtianxu Zhou, Kaiming He, Zeyu Chen, Zihuan Luo, Qiquan Sun

**Affiliations:** 1 Department of Renal Transplantation, Guangdong Provincial People’s Hospital (Guangdong Academy of Medical Sciences), Southern Medical University, Guangzhou, China; 2 Guangdong Cardiovascular Institute, Guangdong Provincial People’s Hospital, Guangdong Academy of Medical Sciences, Guangzhou, China

**Keywords:** chronic kidney disease, drug prediction, fibrosis, ISG15, LYZ, machine learning

## Abstract

**Background:**

CKD represents a substantial worldwide health challenge, defined by the gradual development of tubulointerstitial fibrosis and reduced renal performance. Autoimmune-mediated factors are central to this pathological process, yet early diagnostic biomarkers and specific therapeutic targets are scarce. The objective of this research is to systematically pinpoint crucial specific genes that contribute to autoimmune-mediated CKD progression.

**Methods:**

This study integrates transcriptomics, scRNA-seq, and machine learning to identify PTC-specific drivers in autoimmune-mediated CKD. Bulk RNA-seq (GSE180394, GSE104948) and scRNA-seq were analyzed using LASSO, Random Forest, Boruta, and MCODE algorithms to identify biomarkers, with performance validated via ROC, ANN, and nomograms. Nephroseq clarified gene correlations across CKD subtypes, while immune infiltration mapped microenvironmental interactions. An IRI mouse model validated targets and tested quercetin/all-trans retinoic acid (ATRA).

**Results:**

Multi-algorithm integration robustly identified Lysozyme (LYZ) and Interferon-stimulated gene 15 (ISG15) as core diagnostic biomarkers for autoimmune-mediated CKD, yielding an Area Under the Curve of 0.957 in predictive nomograms and high accuracy (up to 97.7%) in ANN models. ISG15 and LYZ were found to be significantly upregulated in various autoimmune-mediated CKD subtypes according to the Nephroseq database analysis. Importantly, there was a strong positive correlation between their expressions (r = 0.5204, p < 0.0001) and a significant negative correlation with the glomerular filtration rate. Injured PTC subpopulations primarily showed upregulation of LYZ and ISG15, as dynamically localized by single-cell transcriptomics. Immune infiltration studies revealed that LYZ had a strong association with activated CD8+ T cells, while ISG15 was associated with activated dendritic cells. *In vivo* validation demonstrated that quercetin and ATRA successfully reversed the overexpression of LYZ and ISG15, and notably reduced renal fibrotic lesions, as shown by the decreased levels of α-SMA, Fibronectin, and Collagen I in IRI mice.

**Conclusion:**

LYZ and ISG15 are important diagnostic markers and collaborative agents in the development of kidney fibrosis in autoimmune-mediated CKD, with a strong correlation to the severity of the disease. Using agents like quercetin and ATRA to target the LYZ or ISG15 axis represents a promising precision medicine approach to slow the progression of CKD.

## Background

Chronic kidney disease (CKD), characterized by persistent structural or functional issues in the kidneys (GFR below 60 mL/min/1.73m² or albuminuria lasting more than 3 months), impacts more than 800 million individuals worldwide and incurs significant yearly economic expenses (1; 2). Although there have been improvements in early detection techniques such as estimated GFR and urinary biomarkers, the current diagnostic tools are not sensitive enough to identify tubular injury, often resulting in delayed intervention until the damage cannot be reversed (3; 4). The death rates among CKD patients are 5 to 10 times higher than those of age-matched controls, stressing the necessity for novel approaches in diagnosis and therapy (5).

Autoimmune kidney diseases are a leading cause of CKD worldwide, driving both new and existing cases (6). This process typically begins with conditions like lupus nephritis and IgA nephropathy. Systemic immune dysfunction triggers autoantibody production, immune complex deposition, and abnormal cellular immunity (7; 8). This leads to persistent glomerular inflammation. Chronic injury follows, causing irreversible structural damage. As the disease advances, interstitial fibrosis worsens and kidney function declines, often resulting in end-stage renal disease (9). Given this aggressive progression, precise immunomodulatory therapies are urgently needed to alter the CKD trajectory.

CKD progresses through dynamic crosstalk among diverse renal cells, a complexity unexplained by single-mechanism theories (10). Proximal tubular cells (PTCs) are central to this process. They make up over 60% of the renal mass and handle solute reabsorption and metabolic adaptation (11). PTCs are highly context-dependent. Depending on the injury type and environment, they shift from repair to aging or cell death (12). Single-cell studies show marked PTC heterogeneity. Subsets like VCAM1+ICAM1+ cells amplify inflammation and senescence-related genes, which worsens fibrosis (13). Damaged PTCs secrete pro-fibrotic factors such as TGF-β and CTGF. This recruits immune cells and drives extracellular matrix accumulation. Conventional methods like bulk RNA-seq and standard biomarkers such as KIM-1 and NGAL miss these dynamics and cannot predict disease progression. Integrated multi-omics approaches are therefore essential to uncover specific PTC targets that regulate the injury-repair balance (14).

This study sets a precedent by utilizing a multi-omics framework to explore CKD mechanisms and detect translational biomarkers. The integration of GEO bulk RNA-seq and scRNA data led us to identify Lysozyme (LYZ) and Interferon-stimulated gene 15 (ISG15) as autoimmune-mediated CKD biomarkers, verified across different cohorts. These genes were localized to PTCs via scRNA-seq analysis, which revealed their dynamic upregulation in the course of fibrosis. Drug repurposing has revealed all-trans retinoic acid (ATRA) and quercetin as innovative treatments that mitigate fibrosis in mouse models of AKI to CKD. LYZ/ISG15 downregulation led to impaired fibroblast activation, yet their therapeutic modulation resulted in better renal pathology. The study bridges computational forecasts with experimental evidence, delivering practical insights for precision nephrology and underlining the translational potential of multi-omics strategies in managing autoimmune-mediated CKD variability.

## Methods

### Data acquisition

Autoimmune-mediated CKD datasets were obtained from the Gene Expression Omnibus database, accessible at https://www.ncbi.nlm.nih.gov/geo/. Training sets included GSE180394 (GPL19983; 36 CKD vs. 9 controls) and GSE104948 (GPL22945; 21 CKD vs. 18 controls). The validation set comprised GSE104948 (GPL24120; 97 CKD vs. 3 controls). Overview of control and CKD samples derived from GEO datasets, detailing the histopathological classification and corresponding sample counts for each cohort could be found in **Supplementary Additional File 1** (**Supplementary Table 1**). Single-cell analyses utilized merged data from GSE183277 and GSE199711 (GPL24676; 3–3 CKD vs. 9–2 controls). Nephroseq database (https://nephroseq.org/) is analyzed in this study for external validation.

### Differential expression analysis

Differential expression analysis was executed on the GSE180394 and GSE104948 (GPL22945) datasets with the ‘limma’ package (v 3.56.2) to identify DEGs between autoimmune-mediated CKD and control samples (15), and DEGs were screened based on P < 0.05 and |log_2_ fold change (FC)| > 1. Subsequently, the ‘ggplot2’ package (v 3.5.2) (16) was utilized to construct a volcano plot for visualizing the DEGs, and the ‘ComplexHeatmap’ package (v 2.16.0) was utilized to create a heatmap of DEGs (17).

### Recognizing and functionally analyzing potential candidate genes

Candidate genes were identified via Venn analysis using the ggvenn package (v0.1.10). We intersected upregulated and downregulated genes across both datasets (18). Overlapping genes were merged and designated as primary candidate markers. Functional enrichment was performed using the clusterProfiler package (v4.8.1). This included Gene Ontology (GO) and KEGG pathway analyses. A significance threshold of P < 0.05 was applied to all tests (19). Protein-level interactions were explored via the STRING database with confidence score threshold of 0.15.

### Feature gene identification

Using the ‘glmnet’ package (v 4.1.8), the initial screening of candidate feature genes for the GSE180394 dataset was performed with least absolute shrinkage and selection operator (LASSO) regression analysis and 10-fold cross-validation, based on earlier obtained candidate genes (20). Furthermore, random forest was employed to screen candidate feature genes using the ‘random Forest’ package (v 4.7.1.2) (21). Furthermore, the candidate feature genes were also screened using Boruta algorithm via the ‘Boruta’ package (v 8.0.0) (22). Simultaneously, candidate feature genes were screened from the PPI network using the MCODE algorithm in ‘Cytoscape’ software (v 3.10.2) (23), with a node score cutoff of 0.2, a degree cutoff of 5, a maximum depth of 100, and a K-Core of 2. A Venn diagram was generated using the ‘ggvenn’ package (v 0.1.10), and genes from the four algorithms were intersected to serve as feature genes (18).

To analyze the expression of feature genes in the GSE180394, GSE104948 (platform GPL22945), and GSE104948 (platform GPL24120) datasets, the Wilcoxon test was used to assess the differences in their expression levels between CKD and control groups across the three datasets (P < 0.05). Genes with significant expression level differences between CKD and control groups, showing consistent trends across the three datasets, were subsequently identified and called candidate key genes.

We assessed the ability of candidate genes to differentiate CKD from control samples. For the GSE180394 and GSE104948 cohorts, ROC curves were plotted. Analyses utilized the pROC package (v1.18.5) (24). For all candidates, AUC values were computed, and genes with an AUC exceeding 0.7 in both datasets were considered genes.

### Establishment and evaluation of a nomogram and backpropagation neural network analysis

We developed a diagnostic nomogram to estimate CKD risk within the GSE180394 cohort. The ‘rms’ package (v6.8.1) was employed to construct the model using key genes (25). Moreover, the ‘pROC’ package (v1.18.5) was used to generate calibration and ROC curves for determining the predictive capability of the nomogram (24). In addition, Precision-Recall (PR) curves were generated and PR-AUC was computed. Bootstrap validation with 100 iterations was implemented to derive corrected AUC values. Ten repetitions of five-fold cross-validation were used to assess model stability. External validation was carried out on independent external datasets (GSE104948-GPL22945).

The ‘neuralnet’ package (v 1.44.2) was subsequently used, with data normalized by the min-max method [0, 1] and the number of hidden layers established at five (26). In the GSE180394 and GSE104948 (platform GPL22945) datasets respectively, key genes were used to construct artificial neural networks (ANNs), and their classification performance was evaluated with confusion matrix and ROC curves.

Based on the training dataset GSE180394, ROC-curve analyses were performed to systematically compare the individual diagnostic performance of LYZ, ISG15 and well-established renal tubular injury markers HAVCR1 and LCN2 (27). A multi-gene combined regression model was further constructed to quantify the incremental predictive value. For the comparison of C-index among combined models, a baseline logistic regression model containing only LYZ and ISG15 was established. Incremental models were subsequently generated by adding HAVCR1 or LCN2 separately, and a full-gene combined model was constructed with all three genes included. Harrell’s C-index was compared across these groups.

### Chromosome, subcellular localization and GeneMANIA

The ‘RCircos’ package (v1.2.2) was utilized to map the locations of important genes on chromosomes by displaying their distribution (28). Moreover, the DeepLoc (https://services.healthtech.dtu.dk/services/DeepLoc-2.0/) was used to analyze the subcellular localization of each key gene. Furthermore, to understand the associations between the key genes and other functionally similar genes, a GeneMANIA network of key genes was established using the GeneMANIA database (http://genemania.org).

### Gene set enrichment analysis and gene set variation analysis

To explore the functional pathways related to key genes in CKD, GSEA of key genes was performed. The Molecular Signatures Database (MSigDB, https://www.gsea-msigdb.org/gsea/msigdb) was the source for the carefully selected reference gene set ‘c2.cp.kegg_legacy.v2025.1.Hs.symbols.gmt’. We calculated Spearman correlation coefficients between each key gene and all other transcripts in the GSE180394 dataset. Genes were subsequently ranked in descending order based on these coefficients. We then performed GSEA using the ‘clusterProfiler’ package (v4.8.1). Statistical significance was defined by |normalized enrichment score (NES)| > 1 and P.adjust < 0.05, Benjamini-Hochberg (19). Additionally, the dataset underwent GSVA using the ‘GSVA’ package (v1.48.2) (29). Pathway variations between CKD and control samples were identified via Wilcoxon tests (P < 0.05).

### Immune infiltration analysis

We characterized the infiltration landscape of 28 immune cell types via the ssGSEA algorithm. This analysis was implemented using the GSVA package (v1.48.2) (29). Wilcoxon tests identified significant differences in cell abundances between CKD and control groups (P < 0.05). Subsequently, we evaluated the associations between key genes and differential immune cells. Spearman correlation analyses were executed using the ‘psych’ package (v2.5.3). Significant linkages required a correlation coefficient |r| > 0.3 (30).

### Construction of molecular regulatory network

To probe the intricacy and multiplicity of the regulation process of key gene expression by constructing molecular regulatory networks, the miRNAs of key genes were predicted using miRWalk (http://mirwalk.umm.uni-heidelberg.de/) and the TargetScan_8.0 (https://www.targetscan.org/vert_80/), common results from both databases were obtained. Subsequently, the lncRNAs targeting the miRNAs were predicted using the miRNet (https://www.mirnet.ca/) and the ENCORI (https://rnasysu.com/encori/), common results from both databases were obtained. The transcription factors (TFs) were forecasted using hTFtarget (https://guolab.wchscu.cn/hTFtarget/#!/) and ChEA3 (https://maayanlab.cloud/chea3/), and the common results from both databases were obtained. Using ‘Cytoscape’ software (v 3.10.2), TF-mRNA and lncRNA-miRNA-mRNA regulatory networks were eventually assembled (23).

### Molecular docking and compound prediction

Using the Drug Signatures Database (DSigDB, https://dsigdb.tanlab.org/DSigDBv1.0/), researchers aimed to identify compounds potentially related to CKD treatment by forecasting links with two major genes (P < 0.05). To visualize the compound-gene network, ‘Cytoscape’ software (v3.10.2) was utilized (23).

To assess the binding potential of compounds to proteins encoded by each significant gene, compounds that were consistently predicted for these key genes and cited in the literature as connected to CKD were chosen for molecular docking. Structures of proteins in three dimensions, connected to the key genes, were retrieved from the Protein Data Bank (https://www.rcsb.org/). The molecular structures of the compounds were optimized and exported in *.mol2 format using ‘PyMOL’ software (v 2.5.4) (31). The interaction between proteins related to the key genes and the compounds was analyzed using ‘AutoDock Vina’ software (v1.1.2) (32), and the docking binding energies were recorded.

### Single cell data quality control and high-variable gene screening

The two single-cell datasets were merged for subsequent single-cell analyses in this study. To verify the accuracy and trustworthiness of the ensuing data analysis, a comprehensive quality control evaluation was conducted on the sample data within the single-cell dataset using the ‘Seurat’ package (v 5.3.0) (33). The following criteria were used to establish quality control: cells with gene expression counts (nFeature_RNA) fewer than 200 or exceeding 4,000 were removed; cells with cellular unique molecular identifiers (nCount_RNA) surpassing 10,000 were excluded; genes expressed in fewer than 3 cells were filtered out; and cells with a percentage of mitochondrial genes (percent_mt) higher than 5% were also removed.

To acquire genes with relatively high intercellular variation coefficients, the data was normalized using the NormalizeData function from the ‘Seurat’ package (v 5.3.0) (33), and then the vst method of the FindVariableFeatures function was used to extract such genes. Additionally, the first 2,000 highly variable genes (HVGs) exhibiting noticeable fluctuations were shown, and the results illustrated the top 10 genes with the highest variation.

### Cell dimension-reduction clustering and annotation

Data scaling for the identified HVGs across all cell samples was performed using the ScaleData function. The JackStrawPlot function was then utilized to determine which principal components were statistically significant (P < 0.05). The results of reducing data dimensionality were visualized at last with the ElbowPlot function.

To quantify the magnitude of batch effects between the two datasets, batch correction was performed on PCA principal components using the Harmony algorithm based on dataset labels.

The FindNeighbors and FindClusters functions were employed for unsupervised clustering analysis to further determine the quantity of cell clusters. To identify cell clusters, a resolution of 0.1 was used, and the clusters were visualized with the UMAP function. Finally, cell clusters obtained from clustering were annotated, with marker genes retrieved from the literature (34).

### Identification of key cells, pseudotiming analysis, and cell communication

Fisher’s exact test, using the annotated cells, was used to analyze the differences in cell proportions between the CKD and control groups, with a significance threshold of P < 0.05. Using the Wilcoxon test, key gene expression differences between the two groups were evaluated (P < 0.05). Finally, cells with more differentially expressed key genes and reported in the literature to play critical roles in CKD were comprehensively selected as key cells.

Using the ‘Monocle’ package (v2.30.1), pseudotiming analysis was carried out (35). Initially, the key cells were clustered by secondary dimension reduction according to the previous steps (resolution = 0.1). Afterward, marker genes were used to annotate the cell clusters. First, the top 2000 highly variable genes within the dataset were screened as baseline features. Differential expression tests were then performed for each cell cluster, and significant differentially expressed genes with q-value < 0.01 were filtered as core features for constructing the differentiation trajectory. The DDRTree algorithm was adopted for dimensionality reduction to project high-dimensional transcriptional data onto a low-dimensional manifold and build a minimum spanning tree. The root node was automatically identified by the orderCells function, which selected the earliest differentiated cell subset as the starting point of pseudotime. Moreover, the expression of important genes in various cell differentiation processes was also displayed.

Using the ‘CellChat’ package (v 1.6.1), ligand-receptor pair analysis was carried out to examine intercellular interactions (36), and intercellular communication networks as well as ligand-receptor interaction probability plots were visualized.

### Metabolic pathway analysis and TFs regulation analysis

To better analyze the metabolic heterogeneity of key cell subclusters, this study used the ‘scMetabolism’ package (v 0.2.1) (37) to model the metabolic pathway activity of single-cell transcriptome data.

To evaluate the TFs activity of key cells, the ‘dorothea’ package (v1.14.1) (38) was used to calculate the TFs activity of key cell clusters, and to determine the specificity scores of TFs in different cells, the ‘AUCell’ package (v1.22.0) was utilized, followed by normalization using Z-scores (39).

### Key gene expression analysis

The Wilcoxon test was used to analyze differences in key gene expression among samples of various subtypes in the GSE104948 (GPL22945) and GSE104948 (GPL24120) datasets, with a significance level of P < 0.05.

### Animals and IRI induced CKD model

Male C57BL/6 mice (6–8 weeks old, n=25) were maintained under SPF conditions and used in accordance with the Guidelines for Ethical Use of Laboratory Animals (GB/T 35892-2018) and approved by the Guangdong Provincial People’s Hospital Animal Care Committee (KY-Z-2022-026-02). Induction was performed in an induction chamber with 4.0% isoflurane delivered in 100% oxygen at a flow rate of 1.0 L/min. After achieving a surgical plane of anesthesia (confirmed by the absence of pedal withdrawal reflex), mice were maintained on 1.5–2.0% isoflurane via a nose cone throughout the surgical procedure. Initially, eight mice were assigned to each group to account for potential perioperative mortality. Due to the severity of the 26 min ischemia protocol, which results in a predictable transition to CKD, several mice succumbed to the procedure or were excluded due to technical issues during sample collection. Ultimately, five mice per group were included in the final analysis. The mice were divided into five groups at random: IRI​ (bilateral renal pedicle clamping for 26 min, core temperature stabilized at 36.5–37 °C), Sham​ (surgery without clamping), Vehicle​ (0.1% DMSO/saline, i.p.), Quercetin​ (20 mg/kg/day, lot No. Q4951, purity >95%, Merck), and ATRA (1mg/kg/day, lot No. 554720, purity >95%, Merck) (40–42). Treatments including vehicle, quercetin and ATRA were given daily by oral at days 1-7, 14, 21 post-IRI. At 28 days after IRI, mice were euthanized under an overdose of sodium pentobarbital (150 mg/kg, intraperitoneally), with kidneys snap-frozen for molecular analysis or fixed in 4% paraformaldehyde for histopathology (43). Death was confirmed by the cessation of heartbeat and respiration. The process of centrifuging blood samples at 3,000 × g for 15 minutes was used to obtain serum.

### Fibrosis assessment

Renal tissues were obtained without perfusion and fixed in 4% paraformaldehyde. Paraffin-embedded kidney sections (4 μm thick) underwent hematoxylin-eosin (H&E) and periodic acid-Schiff (PAS) staining to assess kidney injury, while Sirius Red (SR) and Masson’s Trichrome (MT) stains were used to evaluate fibrosis.

### Immunohistochemistry

Cold saline (0.9%) was used to initially perfuse the renal tissues, which were then fixed in 4% paraformaldehyde. Immunohistochemistry was conducted on 4 μm paraffin sections as previously detailed (44). Fibrosis was evaluated using antibodies targeting collagen I (COL I; RRID: AB_731684), fibronectin (FN; RRID: AB_2262874), and α-smooth muscle actin (α-SMA; RRID: AB_722538).

### Western blotting assay

Renal tissue underwent perfusion with cold saline (0.9%), followed by protein extraction using a Tissue Homogenizer (KZ-II; Servicebio) and centrifugation at 12,000×g for 15 minutes at 4 °C. Following the collection of the supernatant, the concentration of protein was measured using a BCA assay. For Western blotting, 20 μg of protein per lane was separated on 15% SDS-PAGE gels and transferred to 0.45 μm polyvinylidene difluoride membranes (IPVH00010; Millipore) using a semi-dry transfer system. Membranes were probed with primary antibodies: ISG15 (2743; RRID: AB_2126201; CST), LYZ (ab108508; RRID: AB_10861277; Abcam), and GAPDH (D16H11; RRID: AB_11129865; CST), followed by HRP-conjugated secondary antibodies. The images were analyzed with Fiji-ImageJ, version 1.54f, from NIH in Bethesda, USA.

### Histology and immunohistochemistry analysis

Quantitative fibrosis analysis and immunohistochemical (IHC) evaluation were performed using Fiji-ImageJ (ver. 1.54f, NIH, USA). For fibrosis quantification, five high-resolution digital images per renal section (×100 magnification) were acquired using a Leica DMi8 microscope equipped with a Leica ICC50 HD camera. To ensure metadata integrity, images were captured under consistent lighting and saved as TIFF files. The Color Deconvolution plugin (IHC Profiler) was employed in immunostaining analysis to separate DAB (brown) and hematoxylin (blue) signals, particularly for α-SMA and collagen I. Positively stained regions were isolated using threshold-based segmentation, with parameters kept uniform across all images (Hue: 0–255, Saturation: 0–255, Brightness: 50–255). To calculate the percentage of fibrotic area or positively stained cells, the ratio of thresholded pixels to the entire tissue area was used. To ensure minimal observer bias, two independent investigators, who were not informed about the experimental groups, conducted all analyses. The inter-rater reliability was confirmed with an intraclass correlation coefficient above 0.90.

### Clinical analysis

The Nephroseq v5 database (http://v5.nephroseq.org) serves as a central hub for examining the relationships between clinical characteristics and gene expression in kidney disorders. Using this resource, we investigated the connection between hub gene expression and renal disease attributes.

### Statistical analysis

For bioinformatics analyses, R (v4.2.2) was utilized. The Wilcoxon test assessed group differences, with P < 0.05 indicating statistical significance. GraphPad Prism 10.0 or the online platform https://www.bioinformatics.com.cn was used for statistical comparisons of subset frequencies across groups in other analyses. The analysis involved unpaired t-tests for data that followed a normal distribution, as checked by the Shapiro-Wilk test, and Mann-Whitney U tests for data that was non-parametric. A p-value under 0.05 was deemed statistically significant for all comparisons, with exact p-values specified. For differential expression and enrichment analyses, Benjamini-Hochberg false discovery rate (FDR) correction was applied, and adjusted P-values (FDR < 0.05) were considered statistically significant.

## Results

### Integrated identification of DEGs and functional mechanisms in CKD

**Figure 1:**
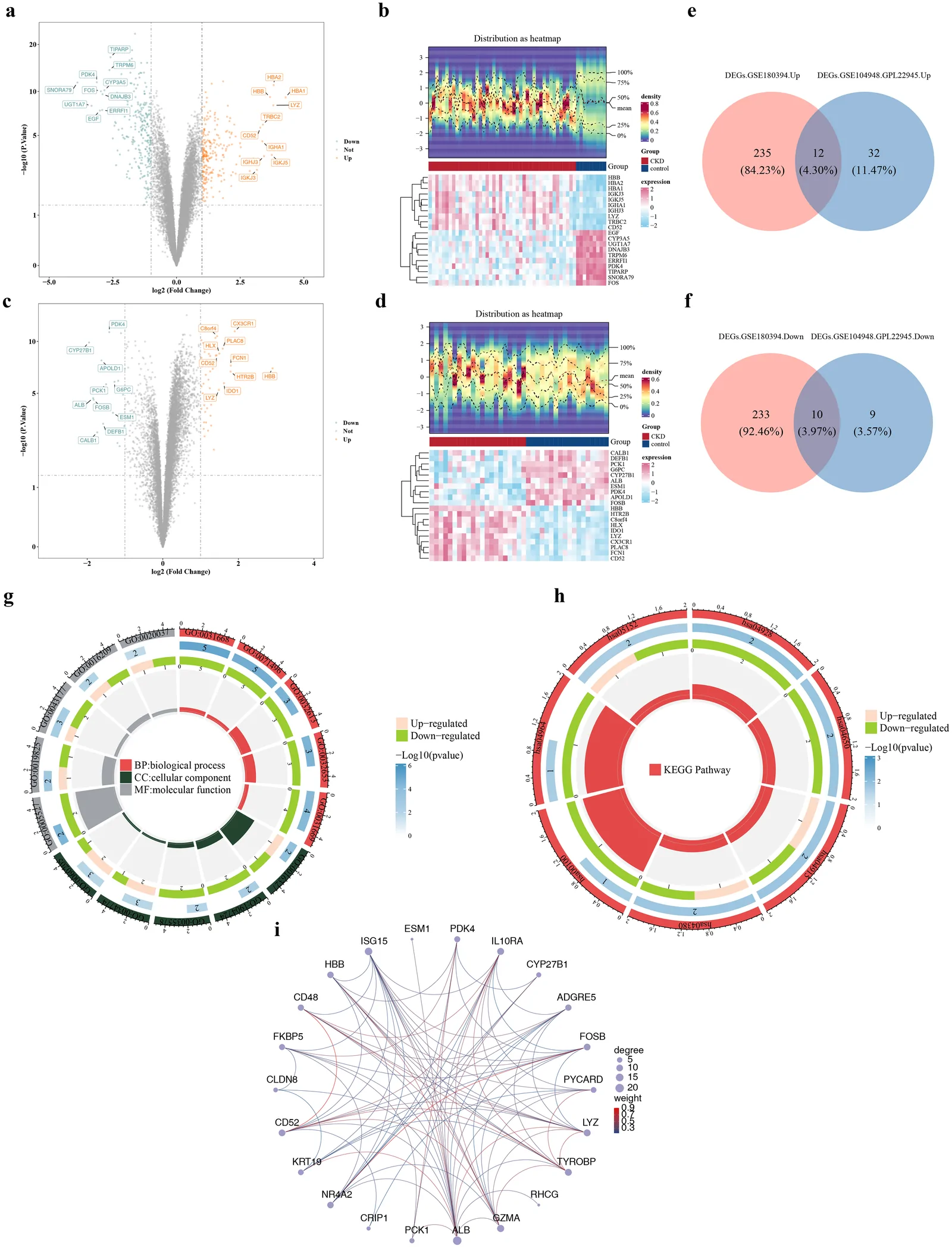
Identification of differentially expressed genes (DEGs) and candidate genes in autoimmune-mediated CKD. **(a, b)** Volcano plot and heatmap illustrating the DEGs in the GSE180394 dataset. **(c, d)** Volcano plot and heatmap showing the DEGs in the GSE104948 (GPL22945) dataset. **(e, f)** Venn diagrams demonstrating the intersection of upregulated and downregulated candidate genes across datasets. **(g)** Gene Ontology (GO) functional enrichment analysis of the overlapping candidate genes. **(h)** Kyoto Encyclopedia of Genes and Genomes (KEGG) pathway enrichment analysis. **(i)** Protein-protein interaction (PPI) network constructed from the candidate genes.

A total of 490 DEGs were identified in the GSE180394 dataset (CKD vs. control), with 247 upregulated and 243 downregulated, while the GSE104948 (GPL22945) dataset identified 63 DEGs, with 44 upregulated and 19 downregulated (**Figures 1a–d**; **Supplementary Additional File 2**). The two datasets’ overlapping analysis uncovered 22 common DEGs, with 12 upregulated and 10 downregulated, which were identified as candidate genes for subsequent research (**Figures 1e, f**).

The candidate genes’ functional enrichment analysis demonstrated significant enrichment of GO terms (P<0.05), producing 327 terms sorted into biological processes (cellular response to extracellular stimulus, interleukin-10 production, nutrient level response), cellular components (apicolateral plasma membrane, tertiary granule lumen, secretory granule lumen), and molecular functions (macrolide binding, oxygen binding, organic acid binding). (**Figure 1g**; **Supplementary Additional File 3**). The analysis of KEGG pathways highlighted seven enriched signaling pathways (P<0.05), which include natural killer cell-mediated cytotoxicity, estrogen signaling, and steroid biosynthesis (**Figure 1h**; **Supplementary Additional File 4**).

In the PPI network analysis, 22 proteins were found to interact, with albumin directly connecting to keratin 19, LYZ, and the beta subunit of hemoglobin (**Figure 1i**). Overall, the prioritized candidate genes highlighted three main functional clusters—immune regulation, interaction with the cell microenvironment, and substance binding/transport—as key mechanisms in autoimmune-mediated CKD pathogenesis. These outcomes establish a foundation for the exploration of mechanisms and the discovery of therapeutic targets.

### Integrated multi-algorithm screening reveals LYZ/ISG15 as key biomarkers for autoimmune-mediated CKD diagnosis and prognosis

**Figure 2:**
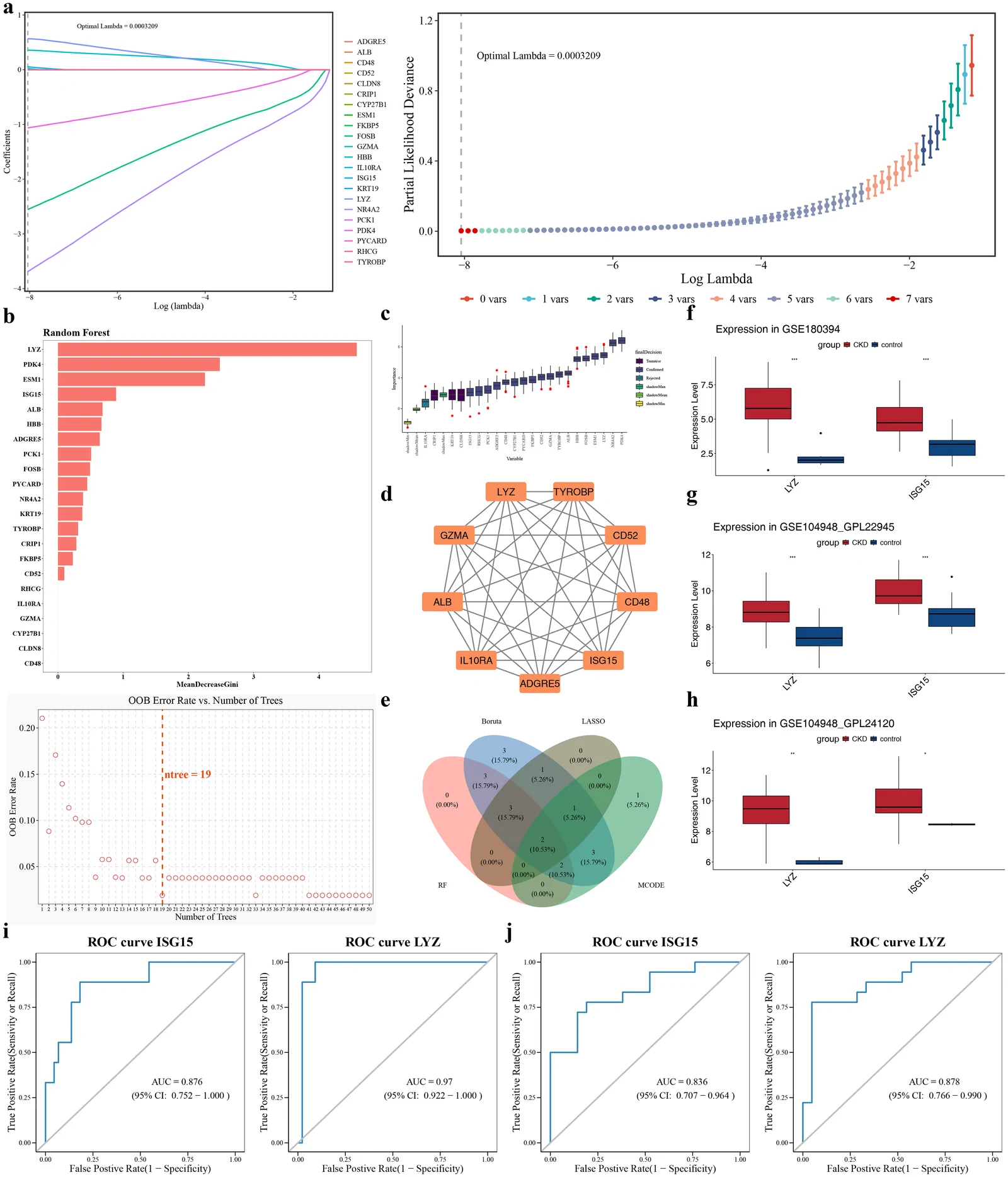
Machine learning-based identification and cross-cohort validation of core diagnostic biomarkers. **(a)** Feature selection using Least Absolute Shrinkage and Selection Operator (LASSO) regression analysis. **(b)** Feature importance ranking utilizing the Random Forest (RF) algorithm. **(c)** Feature selection based on the Boruta algorithm. **(d)** Identification of core functional sub-networks using the MCODE plugin. **(e)** Venn diagram displaying the intersection of feature genes identified by the four machine learning algorithms. **(f–h)** Validation of *LYZ* and *ISG15* expression levels in the GSE180394, GSE104948 (GPL22945), and GSE104948 (GPL24120) datasets. **(i–l)** Receiver Operating Characteristic (ROC) curves assessing the diagnostic performance of *ISG15* and *LYZ* in the GSE180394 and GSE104948 (GPL22945) datasets. *P < 0.05, ***P < 0.001.

Using a multi-algorithm approach, LYZ and ISG15 were identified as primary candidate genes: LASSO regression (λmin= 0.0003209) resulted in 7 candidates (**Figure 2a**), random forest (19 decision trees) identified 10 leading genes (**Figure 2b**), the Boruta algorithm filtered out 18 significant genes (**Figure 2c**), and MCODE detected 9 network-associated genes (**Figure 2d**). These four methods intersected to reveal two overlapping genes (LYZ, ISG15) (**Figure 2e**). Validation in datasets GSE180394, GSE104948 (GPL22945), and GSE104948 (GPL24120) demonstrated that LYZ/ISG15 were significantly upregulated in CKD compared to controls (P< 0.05) (**Figures 2f–h**). The diagnostic performance assessment demonstrated AUC values above 0.8 for both genes in GSE180394 and GSE104948 (GPL22945) (**Figures 2i, j**), validating their application as CKD biomarkers.

### Diagnostic ability and localization of LYZ and ISG15

**Figure 3:**
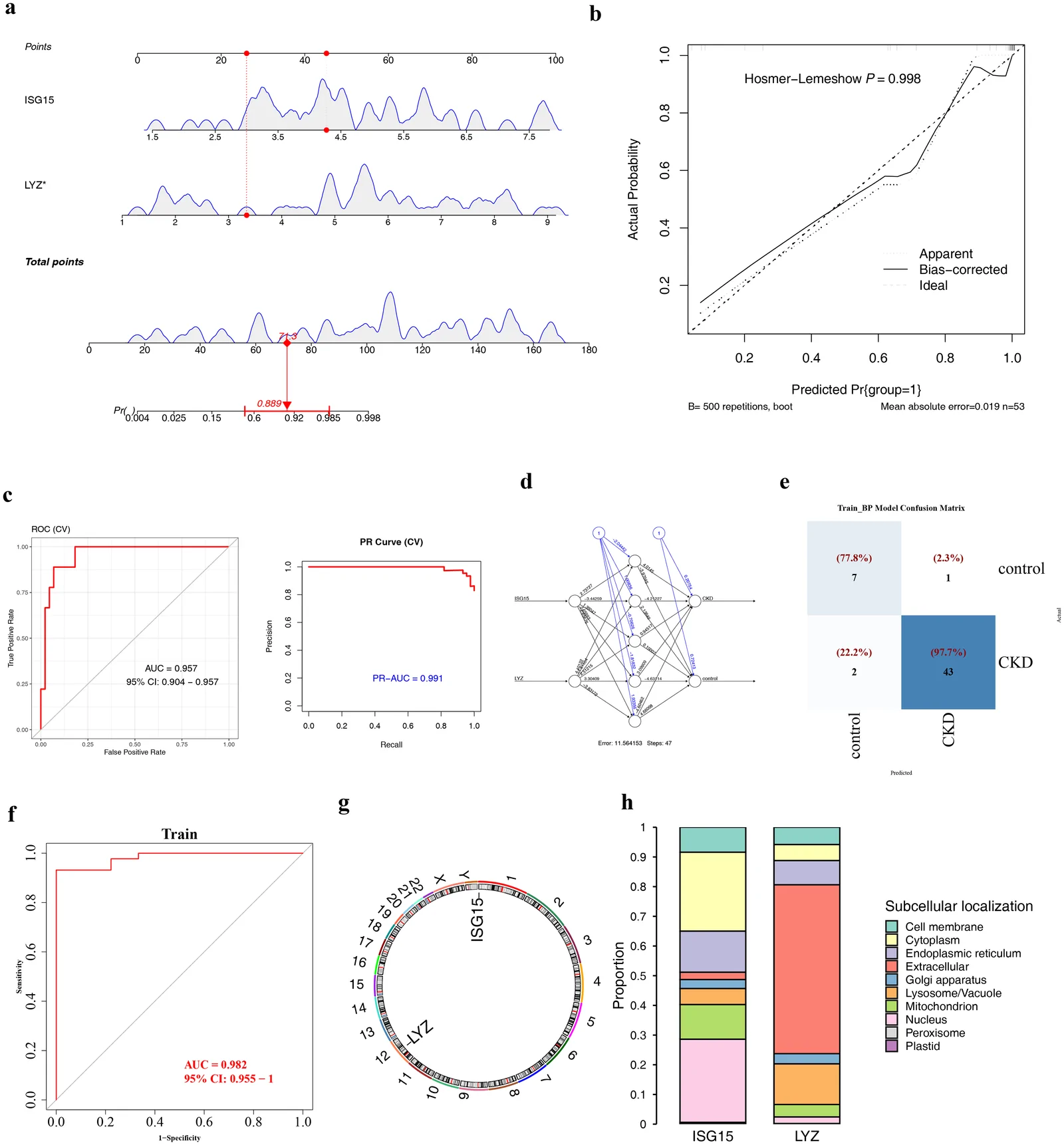
Evaluation of high-dimensional diagnostic models and subcellular localization of LYZ and ISG15. **(a)** Predictive nomogram model constructed by combining *LYZ* and *ISG15* for CKD risk estimation. **(b)** Calibration curve evaluating the predictive accuracy of the nomogram. **(c)** PRROC and ROC curves of the nomogram model. **(d)** Artificial Neural Network (ANN) established in the GSE180394 dataset. **(e)** Confusion matrices assessing the predictive accuracy of the ANN models. **(f)** ROC curve for the GSE180394 ANN model. **(g)** Chromosomal mapping of the key genes. **(h)** Predicted subcellular localization of the LYZ (extracellular) and ISG15 (nuclear) proteins.

A nomogram that integrates LYZ and ISG15 was designed for predicting clinical autoimmune mediated CKD risk (**Figure 3a**), showing excellent performance with calibration curves having slopes close to 1 (P = 0.998) (**Figure 3b**). In addition, the internally validated AUC of the training set after Bootstrap correction was 0.978. The average AUC from ten repeated five-fold cross-validation reached 0.957, with a sensitivity of 0.932, specificity of 0.889, accuracy of 0.925, F1-score of 0.953, and PR-AUC of 0.991 (**Figure 3c**; **Supplementary Additional File 5**). For another training set GSE104948(GPL22945), the AUC was 0.905, sensitivity 0.952, specificity 0.778, accuracy 0.872, F1-score 0.889, and PR-AUC 0.909 (**Supplementary Additional Files 5**, **6**). The ANNs constructed for the datasets GSE180394 and GSE104948 (GPL22945) (**Figure 3d**; **Supplementary Additional File 6**) reached diagnostic accuracies of 97.7% (GSE180394, **Figure 3e**) and 85.7% (GSE104948, **Supplementary Additional File 6**). In the training set, the ANN model yielded an AUC of 0.982 (**Figure 3f**). The AUC reached 0.910 in the validation set GSE104948 (GPL22945) (**Supplementary Additional File 6**). The results demonstrated that the diagnostic model constructed based on LYZ and ISG15 exhibited favorable discriminative ability and generalization performance in the training set, cross-validation, and external validation.

ROC-curve analyses for single-gene models of LYZ, ISG15, HAVCR1 and LCN2 revealed that the AUC values of the two key genes in the present study were higher than those of the known markers (**Supplementary Additional File 6**). These findings partly suggested that their individual diagnostic performance for discriminating CKD and control samples might be superior to that of existing classical renal tubular injury markers.

The multi-gene combined regression model results showed that the baseline LYZ-plus-ISG15 model yielded a C-index of 0.982. Additional incorporation of LCN2 slightly increased the C-index to 0.987, whereas the C-index remained unchanged after adding HAVCR1 (**Supplementary Additional File 6**).

According to chromosomal mapping, ISG15 is found on chromosome 1, while LYZ is on chromosome 12, with both being autosomal (**Figure 3g**). Subcellular localization revealed that LYZ is primarily outside the cell, and ISG15 is within the nucleus (**Figure 3h**). These localization characteristics offer essential starting points for examining autoimmune-mediated CKD regulatory processes and direct future investigations of gene interactions and pathway interactions, connecting genomic context to functional understanding.

### Network analysis, immune modulation, and therapeutic target discovery identifies LYZ/ISG15 as therapeutic targets in autoimmune-mediated CKD

**Figure 4:**
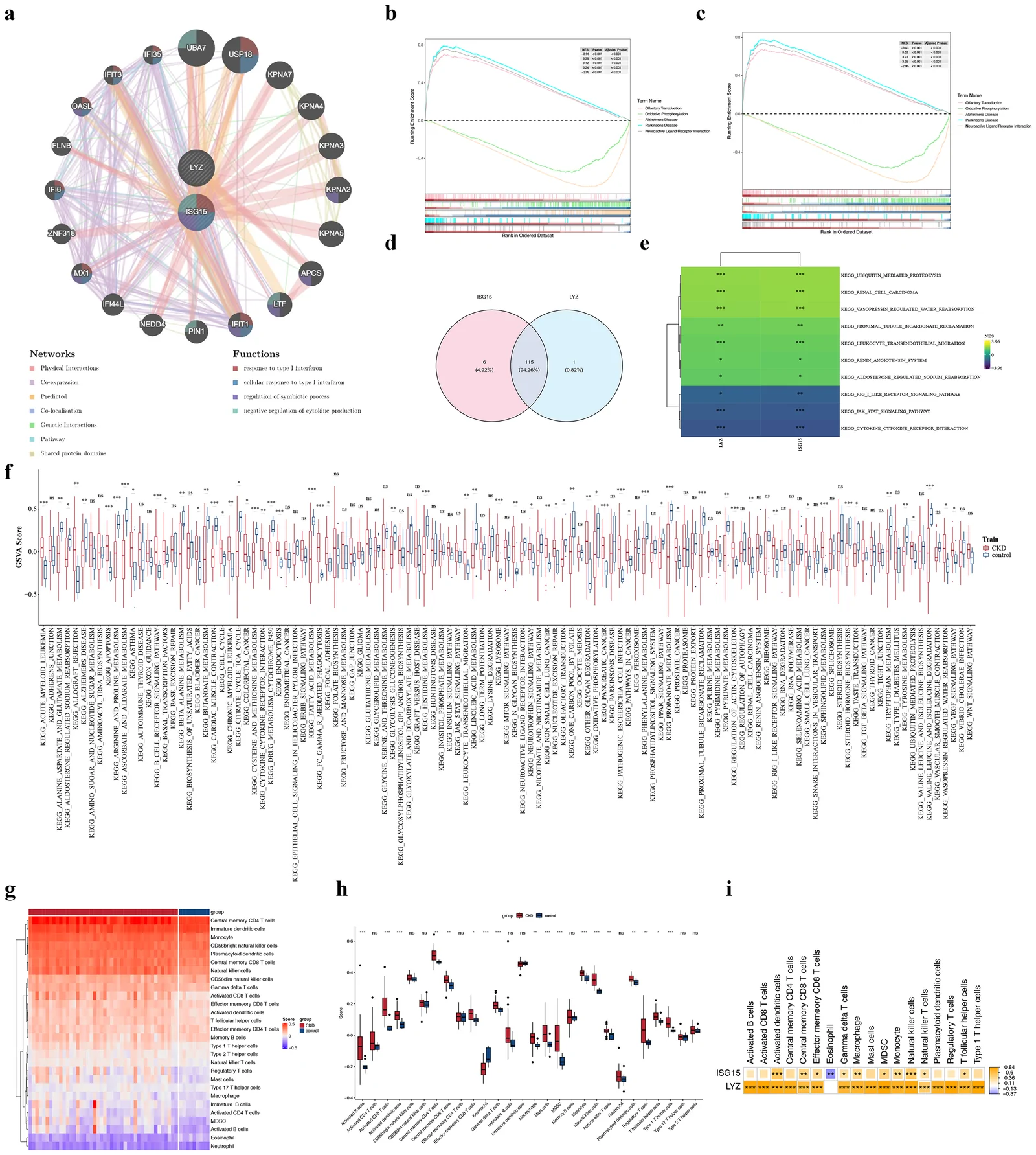
Functional enrichment and immune infiltration analysis. **(a)** GeneMANIA interaction network showing LYZ and ISG15 co-expression. **(b–e)** GSEA profiles for inflammation and JAK-STAT signaling modulated by LYZ and ISG15. **(f)** GSVA identifies key pathway dysregulation within the autoimmune-mediated CKD environment. **(g)** Heatmap showing the relative abundance of 28 immune cell subtypes. Estimates were calculated using the ssGSEA algorithm. **(h)** Differential immune cell infiltration in autoimmune-mediated CKD patients versus healthy controls. **(i)** Correlation matrix between key genes and immune populations.

GeneMANIA network analysis revealed interactions of LYZ, ISG15 with USP18, MX1, and OASL, enriching in type I interferon response, symbiotic process regulation, and negative cytokine production regulation (**Figure 4a**). GSEA showed LYZ enriched in 116 pathways and ISG15 in 121 pathways (**Figures 4b, c**; **Supplementary Additional Files 7**, **8**). They shared 115 common pathways (**Figure 4d**), with top 5 enriched pathways (olfactory transduction, oxidative phosphorylation, neuroactive ligand receptor interaction) identical. Ubiquitin-mediated proteolysis and vasopressin-regulated water reabsorption were positively correlated with both genes, while RIG-I-like receptor and JAK-STAT signaling pathways were negatively correlated (**Figure 4e**).

GSVA identified 61 pathways with significant differences between CKD and control groups (**Figure 4f**). These included leukocyte transendothelial migration, cytokine-receptor interactions, and apoptosis. Immune profiling of 28 subsets revealed 18 differential populations (**Figure 4g**). Specifically, CKD samples showed increased macrophage infiltration and decreased eosinophil proportions (**Figure 4h**). ISG15 displayed the strongest positive correlation with activated dendritic cells (r=0.53, P<0.001). Conversely, it correlated inversely with eosinophils (r=-0.37, P<0.01). LYZ expression was most robustly associated with activated CD8+ T cells (r=0.84, P<0.001; **Figure 4i**; **Supplementary Additional File 9**).

### Multi-omics integration and molecular docking unveil ISG15/LYZ regulatory networks and therapeutic candidates in autoimmune-mediated CKD

**Figure 5:**
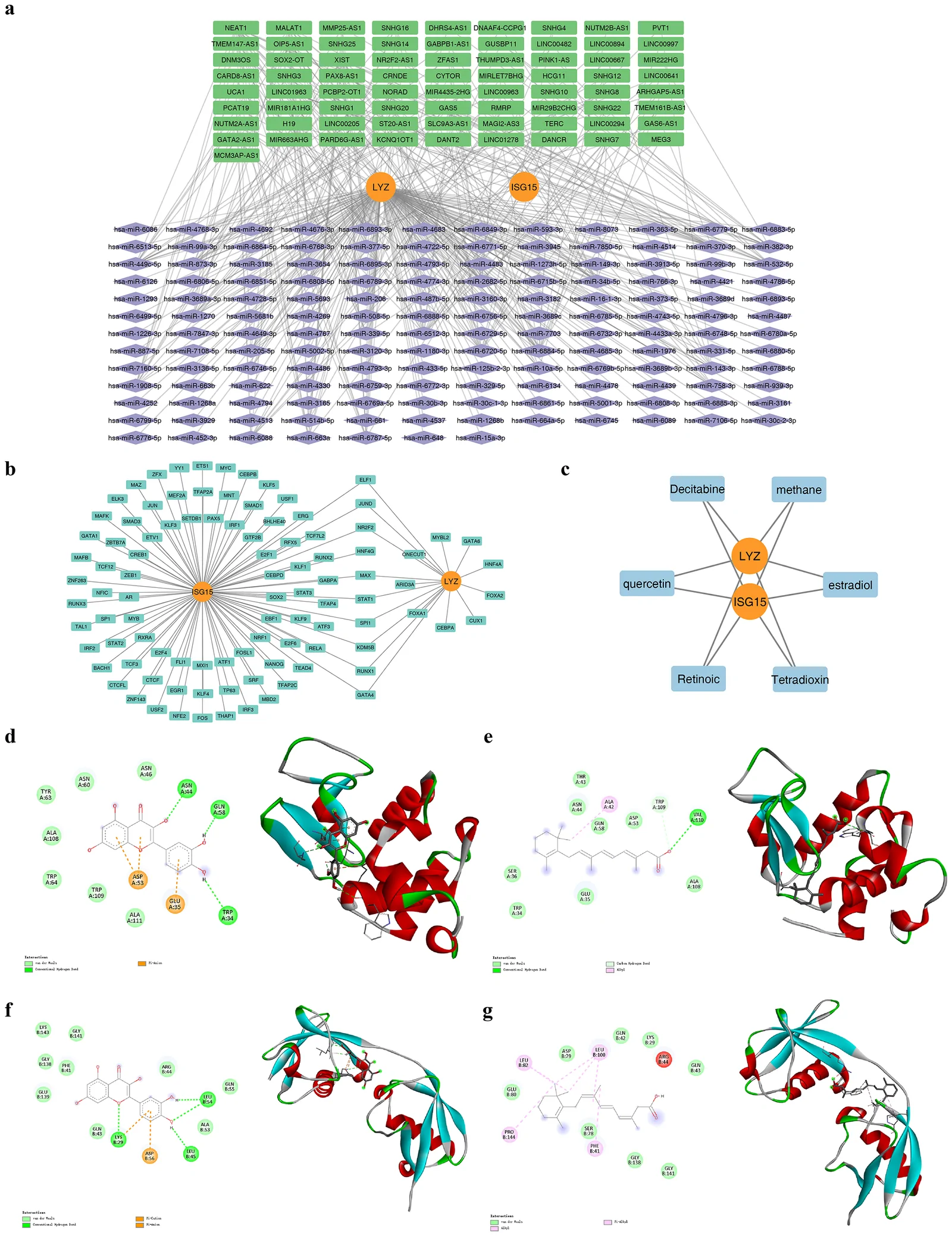
Regulatory networks and molecular docking of therapeutic candidates. **(a)** The lncRNA-miRNA-mRNA network reveals indirect regulation of ISG15 and LYZ. Specific lncRNAs, including SLC9A3-AS1 and SNHG7, modulate this axis via miRNAs. **(b)** TF-mRNA network identifying upstream transcriptional regulators. Ten shared TFs, such as ELF and JUND, co-regulate the key genes. **(c)** Drug-gene interaction network derived from the DSigDB database. **(d–g)** Molecular docking of top candidates, including Quercetin and Retinoic acid.

The lncRNA-miRNA-mRNA regulatory network revealed 3 miRNAs (e.g., hsa-miR-370-3p, hsa-miR-6893-3p) regulating ISG15 and 150 miRNAs (e.g., hsa-miR-3185, hsa-miR-3160-3p) regulating LYZ. A total of 73 lncRNAs (including SLC9A3-AS1, SNHG7, NEAT1) indirectly modulated key gene expression by targeting these miRNAs (**Figure 5a**). The TF-mRNA network identified 98 TFs regulating key genes, with 10 co-regulating both ISG15 and LYZ (e.g., ELF, JUND, NR2F2) (**Figure 5b**), delineating a multi-level regulatory architecture.

Drug prediction via DSigDB identified 33 compounds for ISG15(e.g., decitabine, retinoic acid), 113 for LYZ (e.g., 5-azacytidine, acrylamide), and 6 shared compounds (e.g., retinoic acid, quercetin) (**Figure 5c**; **Supplementary Additional File 10**). Molecular docking showed quercetin had the highest binding energy (-6.6 kcal/mol) with both ISG15 and LYZ (**Table 1**), with active moieties infiltrating binding sites and abundant hydrogen bond donors/acceptors nearby (**Figures 5d–g**).

**Table 1:** Binding energies between compounds and key genes.

Gene name	Compound name	Docking score (kcal/mol)
ISG15	Retinoic	-6.1
ISG15	quercetin	-6.6
LYZ	Retinoic	-5.7
LYZ	quercetin	-6.6

### Nine major cell clusters were identified in autoimmune-mediated CKD using a standard single-cell workflow

**Figure 6:**
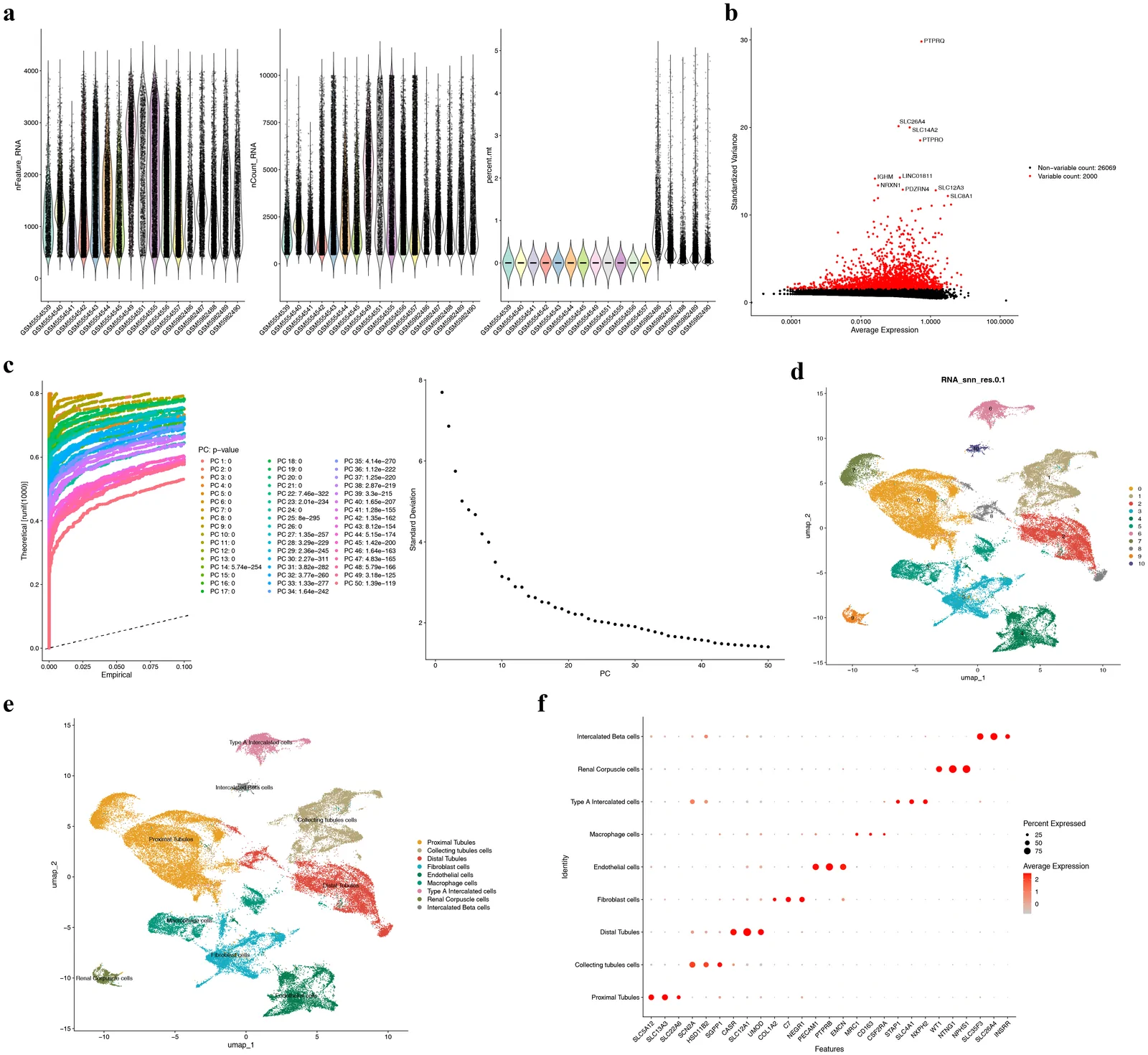
Single-cell RNA-seq quality control and cell type annotation. **(a)** Quality control metrics for merged renal microenvironment datasets. **(b)** Identification of highly variable genes across the cell population. **(c)** Dimensionality reduction via principal component analysis. **(d)** UMAP visualization of 11 distinct cell clusters. **(e, f)** Expression of canonical markers used for cell type annotation. These markers define nine major renal lineages.

We performed scRNA-seq to identify the primary target cells and pathways for LYZ and ISG15. Quality control included filtering cells based on gene counts and mitochondrial content (**Figure 6a**). The analysis identified 2,000 highly variable genes, highlighting top candidates like PTPRQ and SLC26A4 (**Figure 6b**). Based on the elbow plot, 30 significant principal components were selected for clustering (**Figure 6c**). Harmony batch correction validation revealed that the mean cellular homogeneity was 0.825 before correction and 0.778 after correction, corresponding to a decrease of only 5.7%. The silhouette coefficient was 0.00854 prior to correction and 0.00592 after correction, with a difference of merely 0.0026 (**Supplementary Additional Files 11**, **12**). The fluctuations of the two metrics before and after correction were minimal. Furthermore, the silhouette coefficient approached zero after correction, demonstrating that the batch effect between the two original datasets was very weak. Accordingly, the merged raw data obtained via the merge function was directly adopted for subsequent analyses in this study.

Cell clustering process yielded 11 groups, which were annotated into nine major renal cell types (**Figures 6d, e**). Marker gene expression validated these lineages, facilitating subsequent cell-specific investigations (**Figure 6f**; **Supplementary Additional File 13**).

### Single-cell analysis defines stage-specific LYZ/ISG15 dynamics in proximal tubule subsets during autoimmune-mediated CKD progression

**Figure 7:**
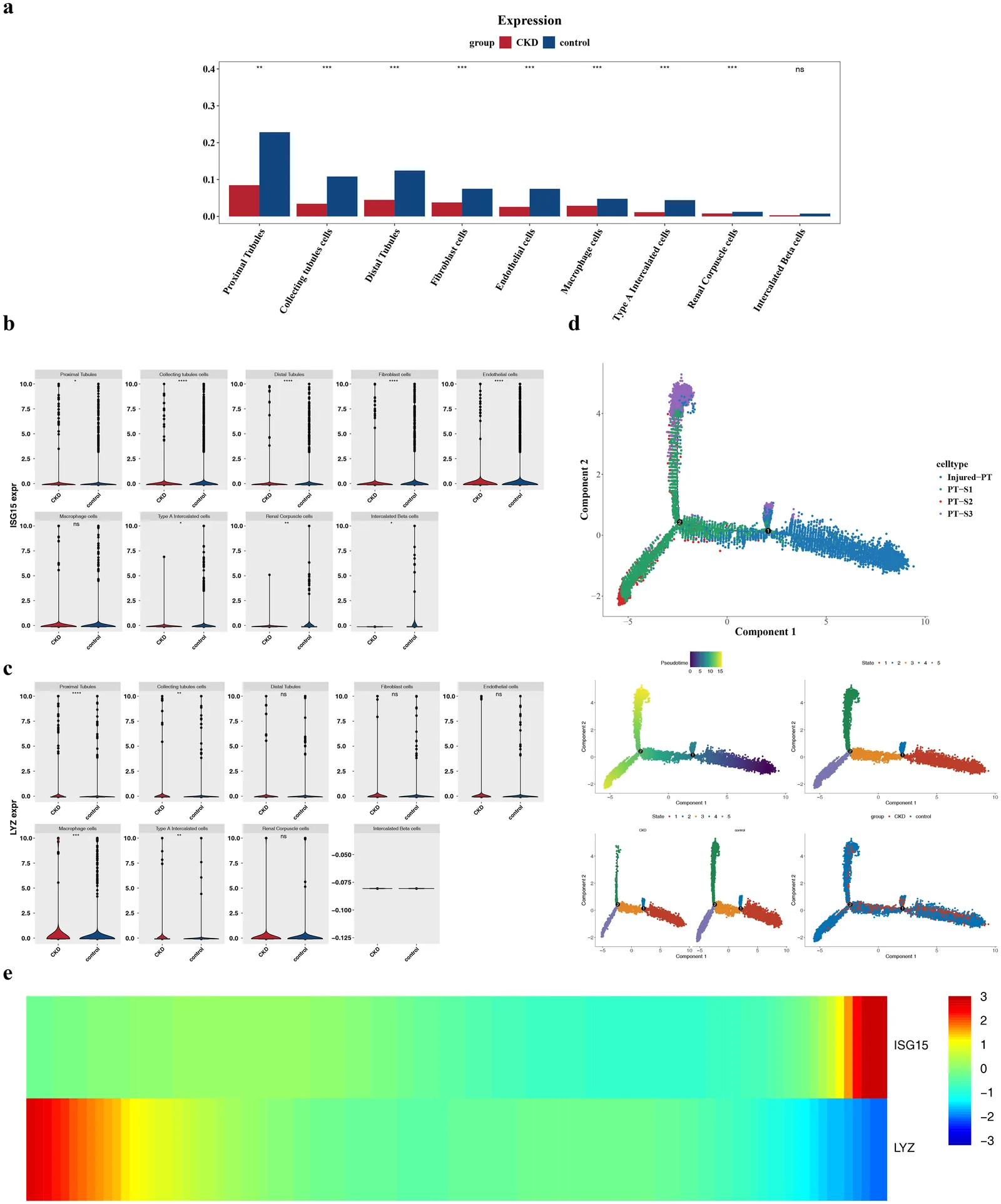
Proximal tubular cell (PTC) heterogeneity and LYZ/ISG15 pseudotime trajectory. **(a)** Cell type proportions in CKD versus control groups. **(b, c)** LYZ and ISG15 expression in injured PTC subsets. Both markers exhibit high specificity for damaged cells. **(d)** Monocle-based developmental trajectory of PTC subclusters. **(e)** Temporal expression of LYZ and ISG15 during PTC differentiation. LYZ characterizes early injury stages. ISG15 expression increases during late-stage remodeling and fibrogenesis. Statistical significance: *P < 0.05, **P < 0.01, ***P < 0.001; ns, non-significant.

Cell type proportion analysis in CKD vs. control samples revealed significant differences in 8 cell types (excluding intercalated beta cells; P<0.01) (**Figure 7a**). LYZ and ISG15 showed differential expression in proximal tubules, collecting tubule cells, and type A intercalated cells (P<0.05) (**Figures 7b, c**). Given the established role of proximal tubules in CKD progression, they were designated as key cells for subsequent analysis.

Re-clustering of proximal tubules identified 4 subtypes (Injured-PT, PT-S1, PT-S2, PT-S3) from 30 principal components (**Supplementary Additional File 14**). Injured-PT represented early differentiation; PT-S1/2/3 represented late differentiation. Differentiation was classified into 5 states (states 1-2: early; states 4-5: late) (**Figure 7d**). ISG15 was highly expressed in late differentiation, while LYZ in early differentiation (**Figure 7e**). These results inform potential pathological mechanisms in CKD.

### Cell communication, metabolic activity, and transcriptional regulation in proximal tubule subtypes of CKD

**Figure 8:**
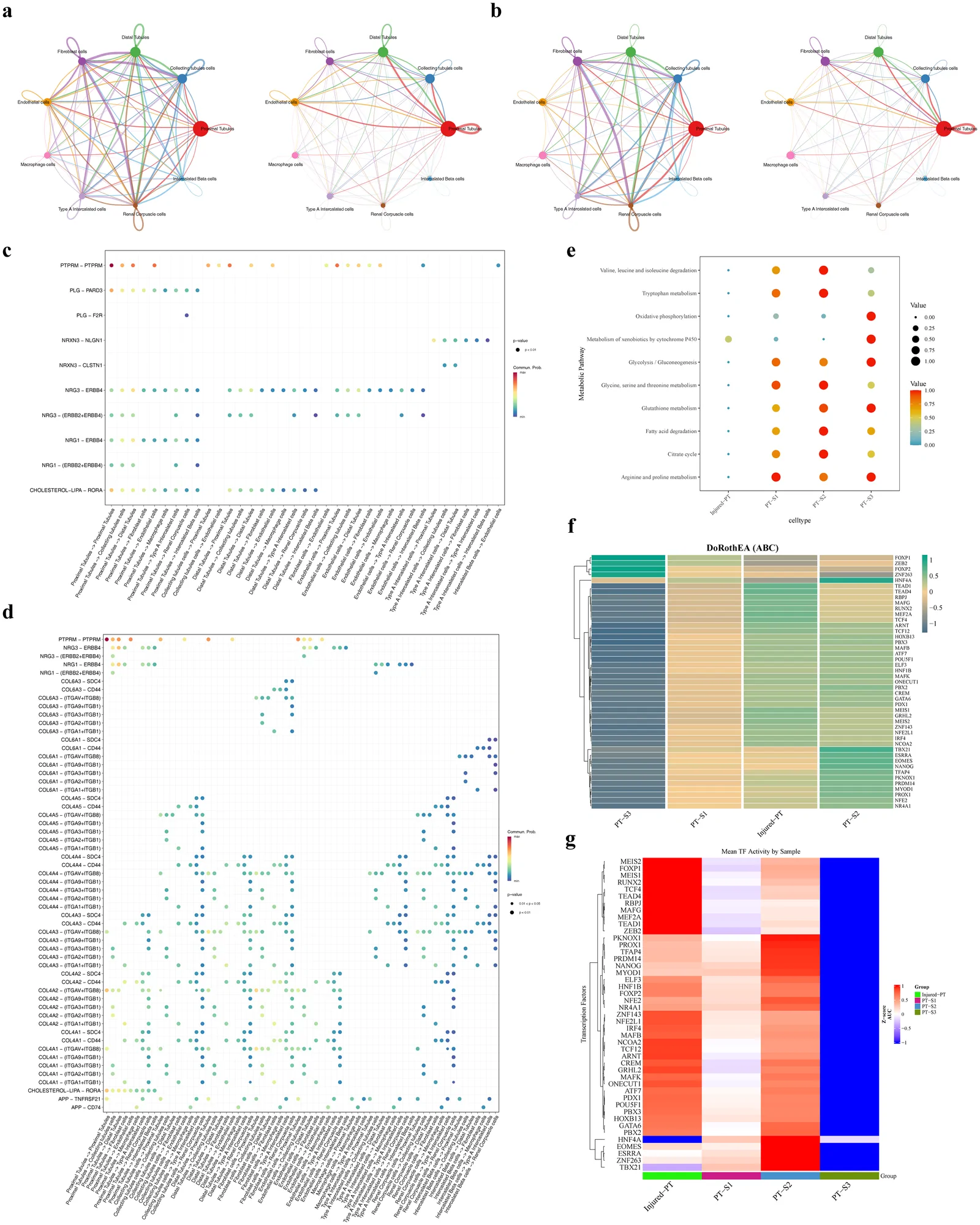
Disrupted cellular communication and metabolic reprogramming in CKD. **(a, b)** Global intercellular communication networks and interaction strengths. Interactions are compared between control and autoimmune-mediated CKD groups. **(c, d)** Significant ligand-receptor interactions across subsets. Notably, PTPRM-PTPRM signaling is disrupted in the CKD microenvironment. **(e)** ScMetabolism analysis of PTC subpopulation metabolic profiles. Significant shifts occur in glycolysis and fatty acid degradation pathways. **(f, g)** Differential activities of key transcription factors in PTC subsets. Analyses were performed using the DoRothEA framework.

Cell communication network analysis revealed reduced communication number and weight between proximal tubules and other cells in CKD versus control samples (**Figures 8a, b**). The study of ligand-receptor interactions indicated that PTPRM-PTPRM showed the most frequent communication in proximal tubule-autonomous signaling across both groups (**Figures 8c, d**). These results emphasize the breakdown of intercellular communication in CKD, suggesting a novel approach for therapies aimed at restoring cell interactions.

Elevated pathway activity, including Glycolysis/Gluconeogenesis, Arginine/proline metabolism, and Fatty acid degradation, was identified in PT-S1, PT-S2, and PT-S3 through cellular metabolic activity analysis (**Figure 8e**). The findings imply that specific metabolic reprogramming by these subpopulations drives CKD pathology, justifying the development of targeted metabolic regulation therapies.

Analysis of transcription factor regulation in key cells predicted 45 TFs, including ARNT, ATF7, and CREM (**Figure 8f**). AUC scores revealed high TFs activity in Injured-PT (e.g., MEIS2, FOXP1, MEIS1) and PT-S2 (e.g., HNF4a, EOMES, ESRRA) (**Figure 8g**).

### Differential expression of LYZ and ISG15 in CKD subtypes

**Figure 9:**
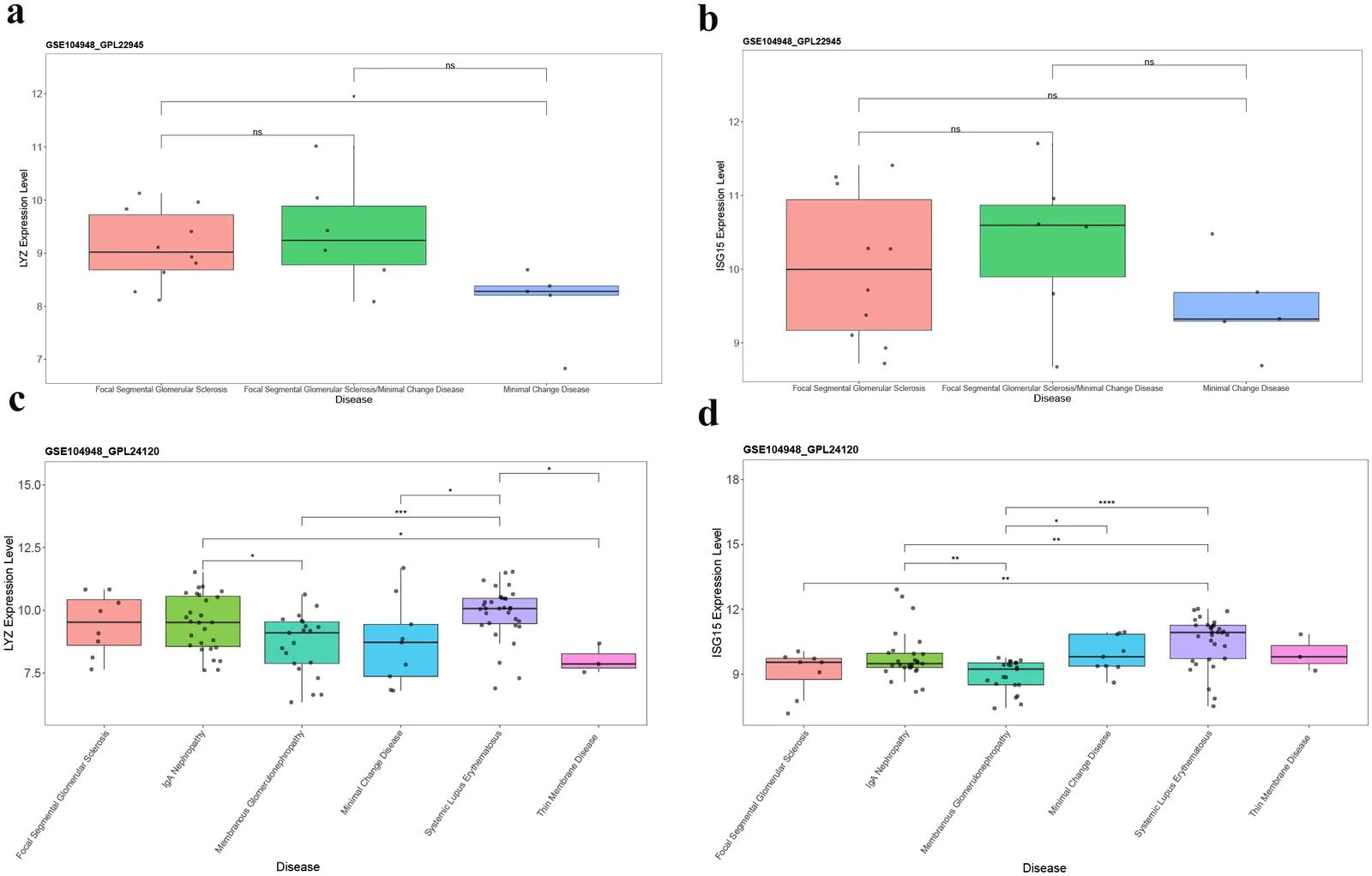
Validation of LYZ and ISG15 expression across clinical autoimmune-mediated CKD etiologies. **(a, b)** Differential expression of LYZ and ISG15 in focal segmental glomerulosclerosis (FSGS) and minimal change disease (MCD). Results are derived from the GSE104948 (GPL22945) dataset. **(c, d)** Expression profiles across an expanded cohort including IgA nephropathy (IgAN), lupus nephritis (SLE), FSGS, membranous glomerulonephropathy, MCD, systemic lupus erythematosus and thin membrane disease. These analyses utilize the GSE104948 (GPL24120) dataset. Statistical significance: *P < 0.05, **P < 0.01, ***P < 0.001; ns, non-significant.

To delve deeper into the specificity of LYZ and ISG15 across various CKDs, we carried out single-cell analysis of CKD with differing origins. The GSE104948 dataset (GPL22945) revealed that LYZ expression was significantly greater in focal segmental glomerulosclerosis (FSGS) compared to minimal change disease (MCD) groups (P < 0.05, **Figure 9a**), with no significant differences among other subtypes (**Figure 9b**). LYZ showed marked expression differences in the GSE104948 dataset (GPL24120) when comparing IgA nephropathy (IgAN) with membranous glomerulopathy (MGN), IgAN with thin basement membrane disease, MGN with systemic lupus erythematosus (SLE), and MCD with SLE (P < 0.05, **Figure 9c**), highlighting subtype-specific regulatory roles.

In the GPL24120 cohort, ISG15 exhibited significant expression differences between FSGS vs. SLE, IgAN vs. MGN, IgAN vs. SLE, MGN vs. MCD, and MGN vs. SLE (P < 0.05, **Figure 9d**).

### Quercetin and ATRA attenuate renal fibrosis by downregulating LYZ and ISG15 in a murine AKI-to-CKD model ​

**Figure 10:**
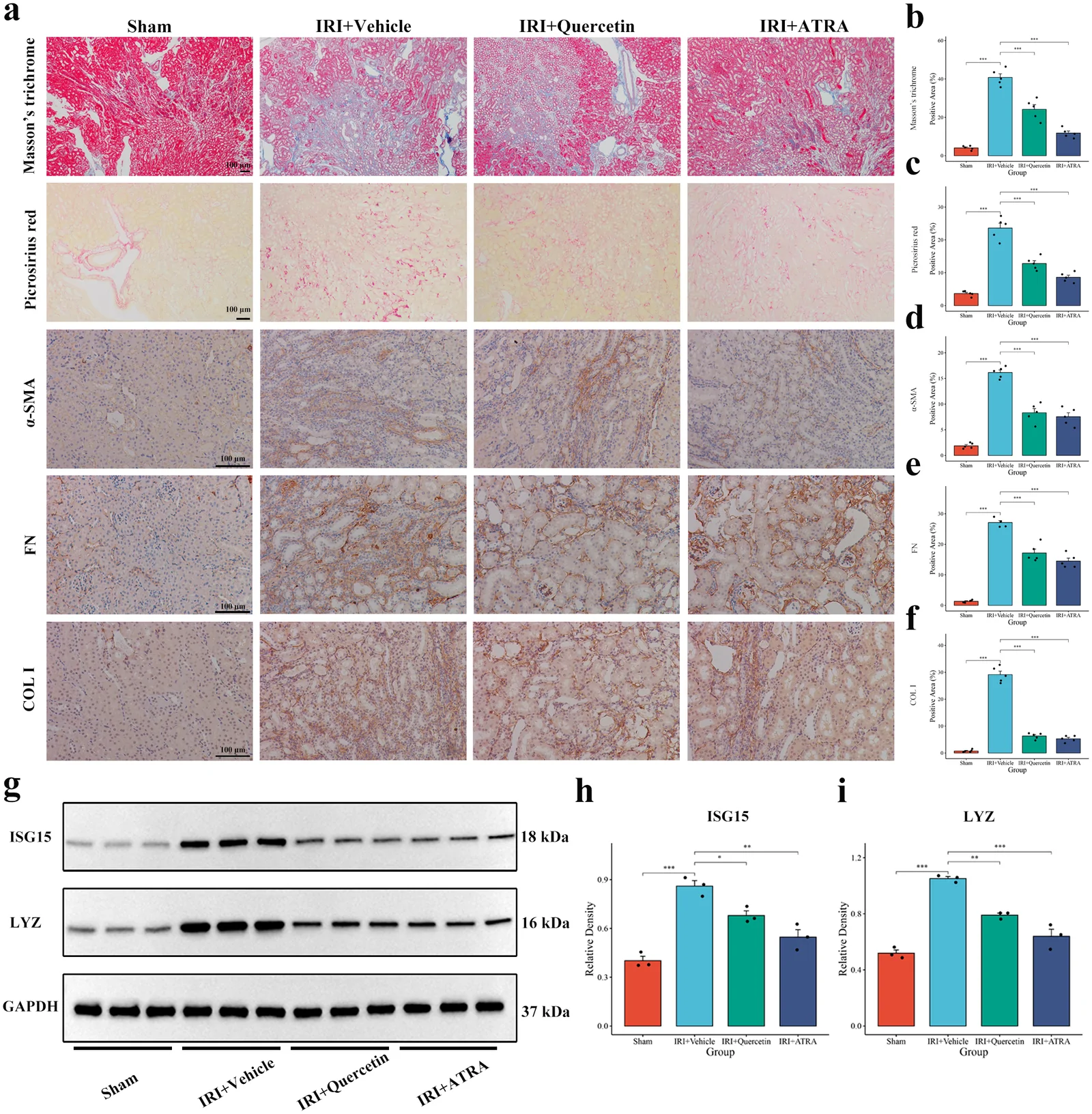
Quercetin and ATRA attenuate renal fibrosis and the LYZ/ISG15 axis *in vivo*. **(a)** Representative histopathology and immunohistochemistry of kidney sections from the four experimental groups. Staining includes Masson’s trichrome, Picrosirius red, α-SMA, FN, and COL I. Sample size is n = 5 mice per group. **(b–f)** ImageJ-based quantification of positively stained fibrotic areas. Both Quercetin and ATRA significantly reduce fibrotic lesions and extracellular matrix deposition. **(g)** Western blot analysis of inflammation markers ISG15 and LYZ in renal tissues. **(h, i)** Densitometric quantification of relative protein expression levels. Quercetin and ATRA treatments significantly suppress IRI-induced ISG15 and LYZ overexpression. Statistical significance: *P < 0.05, **P < 0.01, ***P < 0.001.

We developed a common mouse model to study the transition from AKI to CKD using IRI and assessed the therapeutic impacts of quercetin and ATRA. Representative images from Masson’s trichrome, Picrosirius red, α-SMA, FN, and COL I staining demonstrated that renal fibrosis was notably reduced in mice treated with quercetin or ATRA, as opposed to the IRI + Vehicle group (**Figure 10a**). These changes involved a decrease in collagen deposition, a less extensive fibrotic matrix, and reduced expression of myofibroblast (α-SMA) and extracellular matrix markers (FN and COL I). Further quantitative analysis of these stained sections confirmed that fibrosis-related parameters were significantly reduced in the IRI + Quercetin and IRI + ATRA groups compared to the IRI + Vehicle group (**Figures 10b–f**).

In order to investigate the molecular mechanisms at play, the proteins ISG15 and LYZ, which are associated with inflammation, had their expression measured by Western blot. The blots in **Figure 10g** showed that both ISG15 and LYZ were more expressed in the kidneys of IRI + Vehicle mice than in the sham-operated controls. Importantly, the overexpression of ISG15 and LYZ caused by IRI was effectively diminished by treatment with quercetin or ATRA. **Figures 10h, i** show that quantitative densitometry of Western blot results confirmed a significant reduction in ISG15 and LYZ protein levels in the renal tissues of IRI mice following quercetin and ATRA treatments.

### Nephroseq database analysis reveals distinct expression patterns and clinical correlations of ISG15 and LYZ across autoimmune-mediated CKD ​

**Figure 11:**
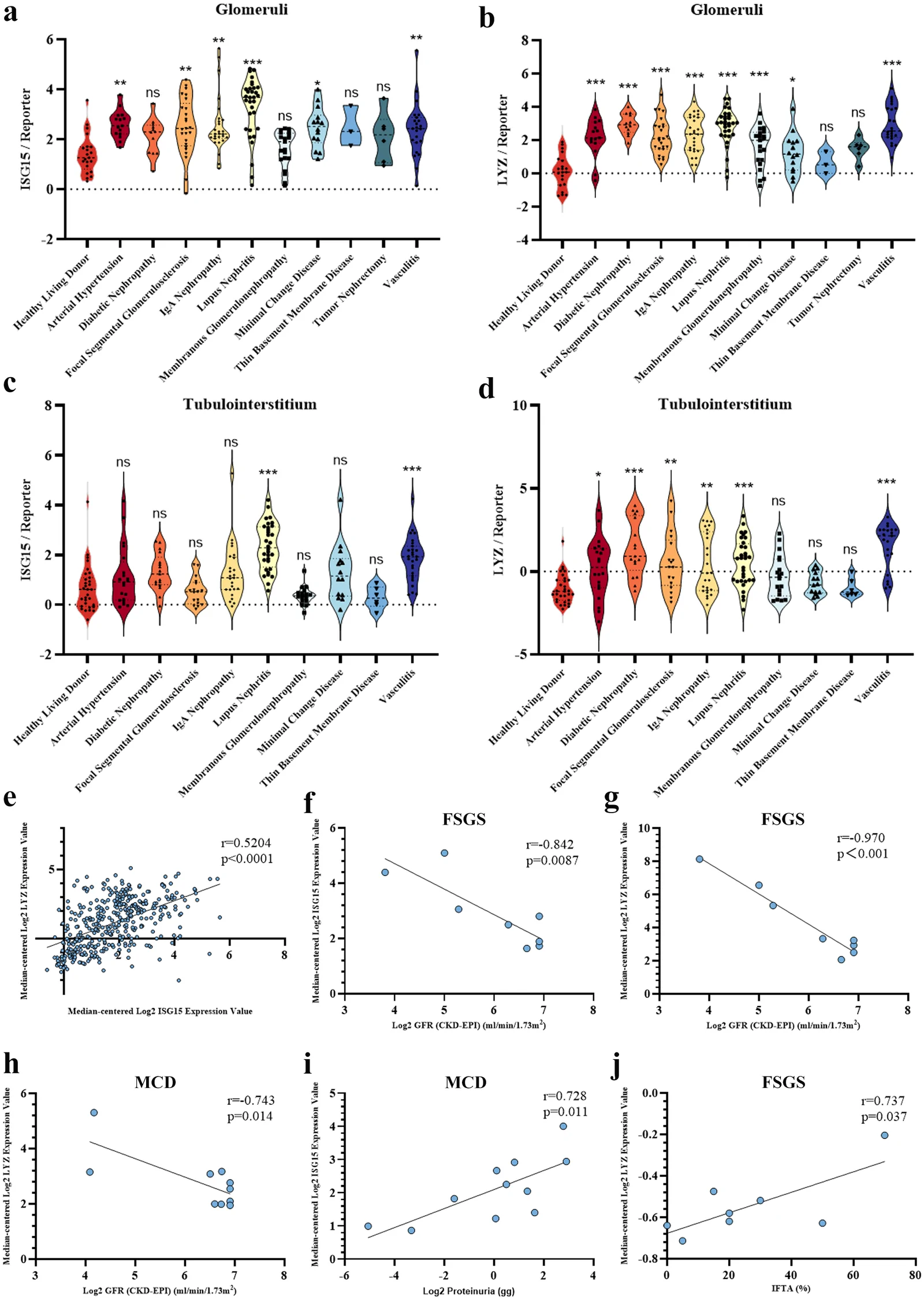
Clinical relevance and Nephroseq-based expression profiles of LYZ and ISG15. **(a–d)** Differential expression of ISG15 and LYZ in glomerular and tubulointerstitial compartments. Levels are compared across multiple kidney diseases. **(e)** LYZ and ISG15 show a significant positive correlation in mixed CKD cohorts. Statistics: r = 0.5204, p < 0.0001. **(f–h)** Negative correlations between gene expression and glomerular filtration rate (GFR). In FSGS, both ISG15 and LYZ correlate inversely with GFR. **(h)** LYZ shows a similar negative correlation in MCD patients. **(i)** Positive association between ISG15 levels and proteinuria severity in MCD. Statistics: r = 0.728, p = 0.011. **(j)** Correlation between LYZ and interstitial fibrosis/tubular atrophy (IFTA) scores in FSGS. Statistics: r = 0.737, p = 0.037. Statistical significance: *P < 0.05, **P < 0.01, ***P < 0.001; ns, non-significant.

Using the Nephroseq database and applying stringent statistical parameters (|r| > 0.7 and p < 0.05), we conducted further analyses to examine the clinical relevance of ISG15 and LYZ in human autoimmune-mediated CKD. Strong correlations were identified between both biomarkers and major clinical parameters across different renal compartments using this approach. The findings indicated that ISG15 is significantly upregulated in the glomeruli of patients with Vasculitis and Lupus Nephritis, and LYZ is consistently elevated in both glomerular and tubulointerstitial regions across different CKD subtypes including FSGS, IgAN, and Lupus Nephritis (**Figures 11a–d**).

Under these more rigorous conditions, the positive association between ISG15 and LYZ expression remained significant (r = 0.5204, p < 0.0001), emphasizing their joint contribution to disease pathogenesis (**Figure 11e**). Moreover, the high-threshold analysis showed even stronger negative correlations between both genes and GFR in FSGS and MCD (**Figures 11f–h**), while also emphasizing more significant positive associations of ISG15 with proteinuria in MCD and LYZ in FSGS (**Figures 11i, j**). The findings, derived from thorough statistical analysis, bolster the reliability of ISG15 and LYZ as biomarkers for monitoring disease progression and kidney function deterioration in autoimmune-mediated CKD, especially FSGS and MCD.

## Discussion

CKD affects over 800 million people globally and is projected to become the fifth leading global cause of death by 2050 (45; 46). Standard indicators including serum creatinine and eGFR lack sufficient sensitivity for early detection, owing to asymptomatic early progression and heterogeneous pathogenesis (47). It is clinically urgent to identify novel mechanism-driven biomarkers for early diagnosis and targeted therapy (48). The study pioneers a combined multi-omics and machine learning approach, addressing a vital gap and establishing a fresh paradigm for autoimmune-mediated CKD research. By changing the diagnostic focus from large-scale systemic indicators to molecular drivers specific to PTC, we decode the complex biological networks of autoimmune-mediated CKD and effectively transform these insights into precision medicine treatments.

Through the integration of bulk RNA-seq and scRNA-seq datasets and the application of four machine learning algorithms, LYZ and ISG15 were identified as core genes in autoimmune-mediated CKD progression. The diagnostic efficacy of these two biomarkers was validated across multiple independent cohorts, demonstrating exceptional accuracy with individual AUC values > 0.8 and a joint diagnostic predictive nomogram AUC reaching 0.957. LYZ and ISG15 reflect a convergent fibrotic signature within an immunologically active microenvironment, regardless of the initiating trigger. Furthermore, we achieved a critical therapeutic breakthrough by demonstrating that pharmacological intervention with quercetin and ATRA remarkably mitigates renal fibrosis during the AKI-to-CKD transition by successfully downregulating the LYZ/ISG15 axis.

LYZ is typically identified as a vital component of the innate immune system, possessing antibacterial features. However, our study and recent evidence highlight a detrimental role of LYZ in autoimmune-mediated CKD progression (49). LYZ is markedly elevated in the tubulointerstitial regions of CKD patients and shows a strong correlation with the presence of immune cells, especially activated CD8+ T cells. Mechanistically, under stress conditions, LYZ is highly secreted by renal fibroblasts and directly promotes their proliferation. Furthermore, activation of the JAK/STAT3 signaling pathway by LYZ is the main cause of renal fibrosis. The induction of a cellular senescence phenotype in renal tubular epithelial cells (RTECs) is also closely related to its overexpression, as evidenced by the significant upregulation of IL-1β and MMP-2 (50). This underscores LYZ as a crucial pro-fibrotic and pro-senescent mediator in the tubulointerstitial microenvironment.

ISG15, which acts as a ubiquitin-like modifier stimulated by interferons, plays a vital role in protein ISGylation and is greatly increased by stress and injury (51; 52). Our single-cell analysis dynamically localized ISG15 to injured PTCs. Recent studies establish that ISG15 acts as a potent accelerator of the AKI to CKD transition. Specifically, ISG15 covalently binds to and ISGylates the type I TGF-β receptor (TGFβR1). This ISGylation competes with and prevents the ubiquitination and subsequent proteasomal degradation of TGFβR1. The pathological stabilization of TGFβR1 leads to the sustained, hyperactive signaling of the TGF-β1/Smad2/3 canonical pathway, culminating in the massive transcription of fibrotic genes such as α-SMA, fibronectin, and collagen (51). Thus, ISG15 serves as a potent intracellular amplifier of profibrotic cascades in injured kidneys.

LYZ and ISG15 outperformed canonical AKI markers (HAVCR1/LCN2) in CKD diagnosis. While the LYZ+ISG15 model achieved high discrimination (C-index), adding LCN2 yielded marginal gains and HAVCR1 none, indicating limited incremental value of acute injury markers in this chronic context. This aligns biologically: HAVCR1/LCN2 reflect acute tubular stress, whereas LYZ/ISG15 mediate chronic interferon-driven inflammation (27).

A striking finding in our study, validated by high-threshold Nephroseq database analysis, is the robust positive correlation between ISG15 and LYZ expression across various CKD subtypes, including FSGS and lupus nephritis. We postulate that LYZ and ISG15 form a synergistic “intra- and extra-cellular” fibrotic loop within the renal microenvironment. ISG15 intracellularly amplifies profibrotic TGF-β/Smad signaling by stabilizing TGFβR1, while secreted LYZ activates JAK/STAT3 to recruit immune cells and trigger tubular senescence (50). Co-activation of these two cascades synergistically drives tubulointerstitial inflammation and irreversible renal fibrosis (53–55).

PTCs are pivotal in maintaining renal homeostasis and orchestrating injury responses via damage-associated molecular patterns that recruit immune cells and activate fibroblasts (56–58). We found concurrent LYZ and ISG15 upregulation in CKD PTCs, correlating with pro-inflammatory cytokine release and ECM deposition. While ISG15 drives mitochondrial dysfunction and pyroptosis via cGAS-STING in diabetic nephropathy (59), LYZ+ PTCs exhibit a glycolytic shift linked to TGF-β1-mediated fibrosis (50). Beyond intrinsic PTC dysfunction, intercellular crosstalk amplifies injury: LYZ+ macrophages secrete CCL2 to recruit fibrogenic monocytes, while ISG15 potentiates TGF-β signaling (60–62). Notably, cell-cell communication analysis identified PTPRM-PTPRM interactions bridging tubular cells and fibroblasts, mechanistically linking LYZ driven inflammation to ISG15 driven fibrosis. This is further reinforced by convergent metabolic reprogramming oxidative phosphorylation in LYZ+ macrophages versus glycolysis in ISG15+ fibroblasts, sustaining the fibrotic niche.

DSigDB screening and molecular docking identified quercetin and ATRA as high-affinity ligands for the LYZ/ISG15 axis. In our IRI-induced CKD model, both compounds significantly attenuated extracellular matrix deposition. Specifically, levels of FN, COL I, and α-SMA decreased following intervention. These treatments also reversed LYZ and ISG15 overexpression *in vivo*. Mechanistically, ATRA confers nephroprotection by inhibiting TGF-β1/Smad3 signaling. It primarily prevents the nuclear translocation of Smad3 (40). Quercetin provides a complementary effect by suppressing endoplasmic reticulum (ER) stress. It downregulates markers such as GRP78 and CHOP while inhibiting the TLR4/NF-κB cascade. This suppression subsequently reduces the release of IL-1β and TNF-α (63). Together, these findings support a dual-target strategy to disrupt the LYZ/ISG15 fibrotic axis.

This study has limitations. First, the molecular crosstalk between LYZ and ISG15 remains undefined; while their causal roles are established, our focus was biomarker identification and pharmacology. Second, single-cell snapshots necessitate longitudinal studies to map temporal dynamics. Third, pooling heterogeneous etiologies may confound the universality of these markers. Fourth, conclusions derive solely from transcriptomics, lacking protein-level spatial validation. Finally, while the IRI model recapitulates fibrosis, it lacks adaptive immune complexity; validation in classical autoimmune models and spatial transcriptomics is warranted (64, 65). While the concurrent downregulation of LYZ/ISG15 and fibrotic markers suggests a mechanistic link, the current study does not provide direct genetic evidence that the therapeutic benefits of quercetin or ATRA occur exclusively via this axis. Future studies utilizing specific antagonists or conditional knockouts are necessary to confirm target specificity.

## Conclusion

In conclusion, integrated multi-omics and machine learning identified LYZ and ISG15 as key autoimmune-mediated CKD biomarkers. These proteins also function as candidate genes of renal fibrosis. Pharmacological targeting of this axis via quercetin or ATRA offers a viable therapeutic strategy. Such precision interventions could effectively arrest CKD progression.

## Data Availability

The datasets presented in this study can be found in online repositories. The names of the repository/repositories and accession number(s) can be found in the article/**Supplementary Material**.

## Supplementary material

Supplementary Figure 1 Validation of the diagnostic performance evaluation. **(a)** PRROC and ROC curves for validation set GSE104948 (GPL22945). **(b)** ANN topologies established in the validation set GSE104948 (GPL22945). **(c)** Confusion matrix for the validation set ANN model. **(d)** ROC curve for the validation set ANN model. **(e)** Single-gene ROC curve. **(f)** The C-index for multigenic joint regression models.

Supplementary Figure 2 UMAP plots before and after batch processing.

Supplementary Figure 3 Dimensionality reduction and cell type annotation of proximal tubular cell (PTC) subtypes. **(a)** Scree plot showing the standard deviations across principal components (PCs) to determine the optimal dimensionality. **(b)** Uniform Manifold Approximation and Projection (UMAP) visualization of unsupervised PTC clustering. **(c)** UMAP plot displaying the final cell type annotations for distinct PTC subclusters. **(d)** Dot plot illustrating the expression profiles of specific marker genes across different clusters. Dot size represents the percentage of cells expressing the gene, and color intensity indicates the average expression level.
