# Supplementary material for: Multi-omics and machine learning reveal LYZ and ISG15 as diagnostic and therapeutic targets in autoimmune-mediated chronic kidney disease
Source: Front Immunol. 2026 Sep 1;17:1922463. doi: 10.3389/fimmu.2026.1922463 (PMC13575749; doi:10.3389/fimmu.2026.1922463)
Supplement: Supplementary file 1 [file Table1.docx]

Table S1. Distribution of pathological subtypes across the integrated GEO datasets

| Datasets | Control group | CKD group |
| --- | --- | --- |
| GSE180394 (GPL19983) | Normal Renal Tissue (9) | Lupus Nephritis (14)  Focal Segmental Glomerulosclerosis (9)  IgA Nephropathy (4)  Interstitial Nephritis (2)  Membranous Nephropathy (2)  Thin Basement Membrane Disease (2)  Chronic Glomerulonephritis with CLL Infiltration (1)  Light Chain Deposition Disease (1)  Minimal Change Disease (1) |
| GSE104948 (GPL22945) | Normal Renal Tissue (18) | Focal Segmental Glomerulosclerosis (10)  Minimal Change Disease (5)  Focal Segmental Glomerulosclerosis /Minimal Change Disease (6) |
| GSE104948 (GPL24120) | Normal Renal Tissue (3) | Lupus Nephritis (32)  IgA Nephropathy (27)  Membranous Glomerulonephritis (21)  Minimal Change Disease (9)  Focal Segmental Glomerulosclerosis (8)  Thin Basement Membrane Disease (3) |
